# Evaluation of Ki-67 expression in feline non-ocular melanocytic tumours

**DOI:** 10.1186/s12917-018-1639-1

**Published:** 2018-10-12

**Authors:** Silvia Sabattini, Andrea Renzi, Francesco Albanese, Marco Fantinati, Antonella Rigillo, Francesca Abramo, Raimondo Tornago, Giovanni Tortorella, Maria Massaro, Teresa Bruna Pagano, Julia Buchholz, Giuliano Bettini

**Affiliations:** 1 Department of Veterinary Medical Sciences, Alma Mater Studiorum University of Bologna, Via Tolara di Sopra, 50, 40064 Ozzano Emilia, (BO) Italy; 2 “La Vallonea” laboratory, Via Giuseppe Sirtori, 9, 20017 Rho, MI) Italy; 3 Department of Veterinary Sciences, University of Pisa, Viale delle Piagge, 1, 56124 Pisa, Italy; 4 “Città di Bolzano” veterinary clinic, Via Resia, 20, 39100 Bolzano, Italy; 5 Radiation Oncology Consultant, Unterrenggstrasse 36, CH-8135 Langnau am Albis, Switzerland

**Keywords:** Feline, Melanoma, Ki-67 index, Proliferative activity, Mitotic count, Prognosis

## Abstract

**Background:**

Melanomas are rare in cats. The eye is the most commonly involved site, whereas few data are available about feline non-ocular melanomas (NOMs). Ki-67 thresholds with prognostic relevance have been established for canine melanomas, but not in cats. This study was undertaken to investigate the relationship between Ki-67 index, tumour characteristics, and clinical outcome in feline NOMs.

Histologic samples were retrospectively reviewed. Amelanotic tumours were admitted upon immunohistochemical positivity for Melan A or S100. Evaluated parameters included morphological diagnosis, histotype, junctional activity, degree of pigmentation, vascular invasion, lymphocytic infiltrate, necrosis, mitotic count (MC) and Ki-67 index. Pigmented tumours were bleached before evaluation. Clinical and follow-up information were retrieved via telephone interviews with the referring veterinarians.

**Results:**

Fifty tumours located in skin (*n* = 33) and mucosae (*n* = 17) were included. Forty-eight percent and 95% of amelanotic tumours (*n* = 21) stained positive for Melan A and S100, respectively. Most achromic tumours were mucosal (*P* < 0.001, Fisher’s exact test) and presented a spindle cell morphology (*P* = 0.002; Fisher’s exact test). MC and Ki-67 index were significantly correlated (*P* < 0.001; *R* = 0.67; Spearman’s rank correlation); median values were 15 (range, 0–153) and 28% (range, 1–78%), respectively. Both were significantly higher in spindle cell melanomas, in tumours lacking junctional activity and in poorly-pigmented tumours. Follow-up information was available for 33 cats (66%). Variables related with a poor clinical outcome included mucosal location, tumour size, spindle, balloon and signet ring cell histotypes, low pigmentation, MC > 5, Ki-67 > 20% and lack of treatment administration. On multivariable analysis, only tumour histotype and treatment retained prognostic significance.

**Conclusions:**

Although the majority of feline NOMs behave aggressively, Ki-67 index, together with other parameters, may contribute to prognostic assessment. Prospective studies on homogeneous populations are warranted to identify reliable threshold values for this marker.

## Background

Non-ocular melanocytic neoplasms (NOMs) are extremely rare in cats, accounting for 2.7% of all skin tumours and less than 1% of oral tumours [1, 2].

The few studies reporting the clinical evolution of NOMs and investigating factors of potential prognostic interest have so far generated conflicting results [1, 3–6]. The most cited parameters associated with a worse outcome include eyelid or mucosal location, achromic phenotype and epithelioid morphology [1, 3, 4, 6] whereas tumours arising on the ear pinna may exhibit a more favourable prognosis [4].

The relative number of tumour cells positive for the nuclear protein Ki-67 (Ki-67 index, tumour growth fraction) is an acknowledged prognostic factor for canine melanoma [7–9]. In this species, the Ki-67 index has been demonstrated to be significantly different between benign and malignant melanocytic neoplasms, and negatively correlated with survival. Consequently, thresholds holding a prognostic value have been established for both cutaneous and oral canine melanocytic neoplasms, and the assessment of the growth fraction has become part of the routine histology practice for these tumours [7–9].

This is the first study investigating the relevance of the growth fraction in feline melanocytic tumours. The primary goal was to evaluate the relationship between Ki-67 index and tumour characteristics, including anatomic location, size, histologic malignancy, predominant histotype and mitotic count. Secondly, we aimed to evaluate whether the Ki-67 index was related to clinical outcome and survival times in a subset of cats with available follow-up information.

## Results

### Demographic information and tumour characteristics

Fifty feline melanocytic tumours fulfilled the inclusion criteria. Breeds included 42 Domestic Shorthairs, 3 Persians, 2 Siamese, 1 Maine Coon, 1 Devon Rex and 1 Chartreux. There were 26 castrated males (52%) and 24 spayed females (48%), with a mean age of 11 ± 4 years (range, 2–19). Information regarding hair colour and living environment were available for 33 cats (66%). Most represented coat colours included grey tabby (*n* = 10), red tabby (*n* = 8), black solid or bicolor black-white (*n* = 5), brown tabby (*n* = 4) and calico (*n* = 4). Cats had outdoor access in 18 out of 33 cases (54%).

Thirty-three tumours (66%) were located in the skin; including ear pinna (*n* = 7), eyelids (*n* = 4), face (*n* = 4), trunk (*n* = 11), limbs (*n =* 3) and digits (*n* = 4). Eleven out of 20 (55%) of these cats had no outdoor access. Seventeen tumours (34%) were in a mucosal location or in a mucocutaneous junction (oral mucosa, *n* = 10; lip, *n* = 6; nasal mucosa, *n* = 1). Median tumour diameter before fixation was 1.3 cm (range, 0.3–4). Regional lymph node metastases had been cytologically or histologically identified at diagnosis in 5 cutaneous melanomas (2 digital and one each on eyelid, ear pinna and trunk).

### Histology and immunohistochemistry

**Fig. 1:**
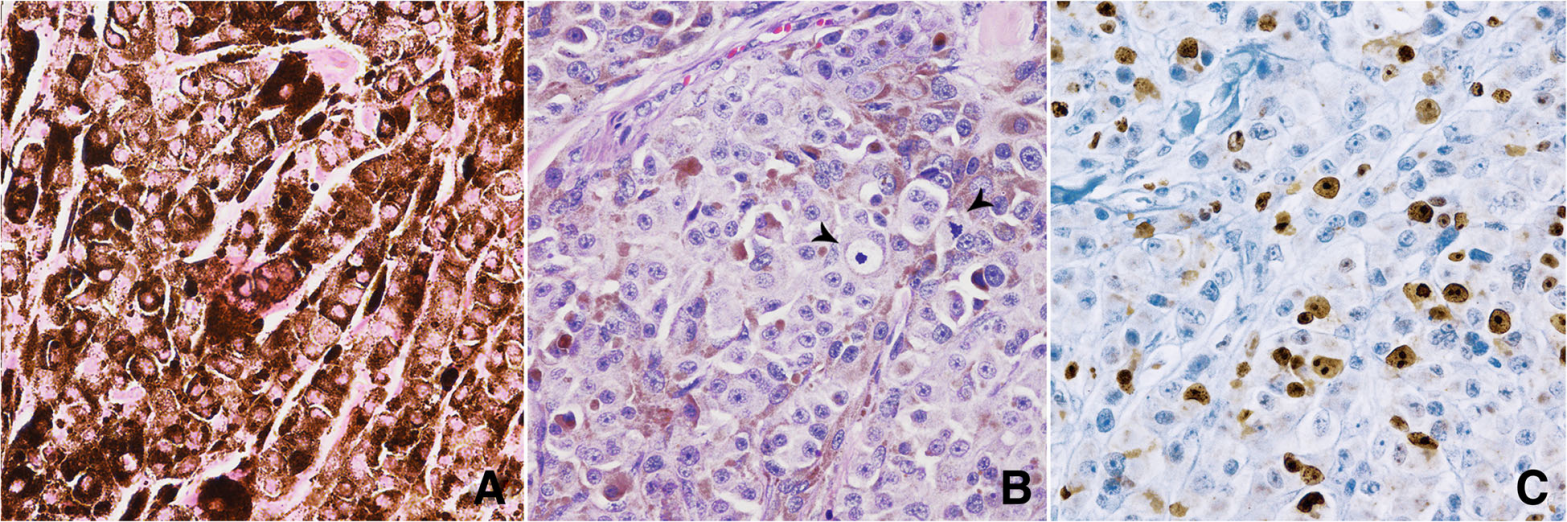
**a** Heavily pigmented epithelioid melanoma. Haematoxylin and Eosin (HE), 400× magnification. **b** The same case after bleaching with potassium permanganate and oxalic acid, showing several mitotic figures (arrowheads). HE, 400× magnification. **c** The same case after bleaching and Ki-67 immunostaining. MIB1 immunohistochemistry (IHC), haematoxylin counterstain, 400× magnification

**Fig. 2:**
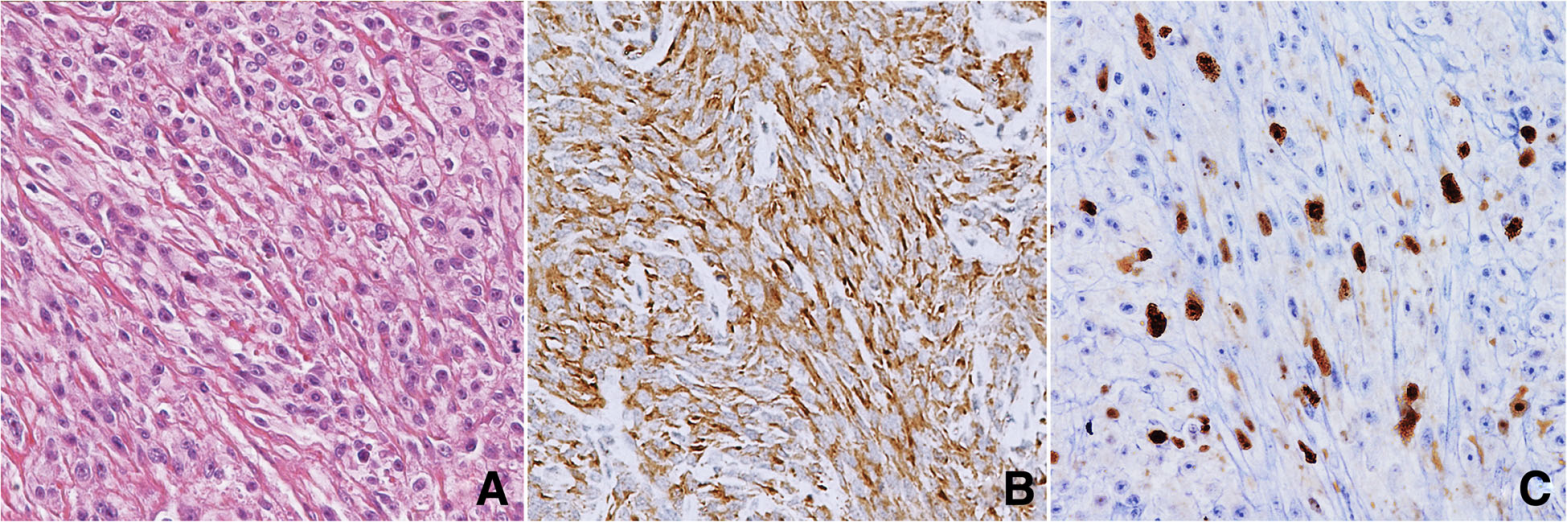
**a** Amelanotic spindle cell melanoma. HE, 400× magnification. **b** The same case after immunostaining for Melan A, demonstrating the melanocytic nature of the tumour. Melan A IHC, haematoxylin counterstain, 200× magnification. **c** The same case after Ki-67 immunostaining. MIB1 IHC, haematoxylin counterstain, 400× magnification

**Fig. 3:**
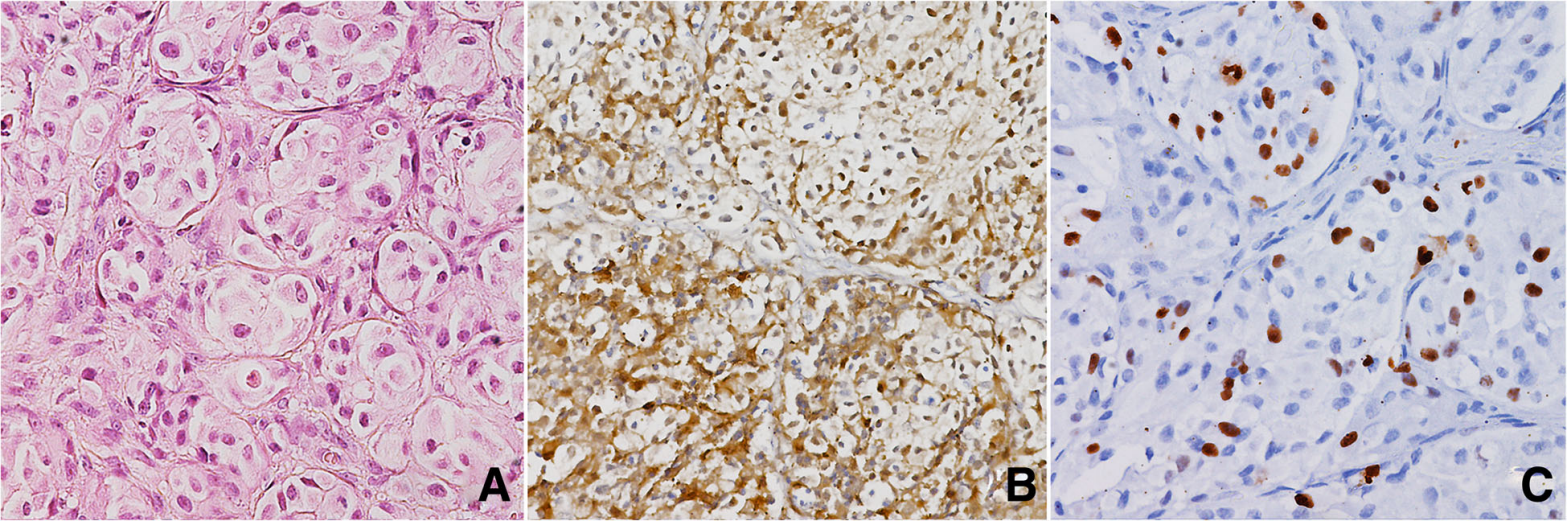
**a** Amelanotic balloon cell melanoma. HE, 400× magnification. **b** The same case after immunostaining for S100, demonstrating the melanocytic nature of the tumour. S100 IHC, haematoxylin counterstain, 200× magnification. **c** The same case after Ki-67 immunostaining. MIB1 IHC, haematoxylin counterstain, 400× magnification

Forty-three cases (86%; 27 cutaneous and 16 mucosal) were diagnosed as malignant melanomas and 7 (14%; 6 cutaneous and 1 oral) as melanocytomas. According to the prevalent histotype, there were 12 epithelioid, 8 spindle cell, 5 balloon cell, 1 signet ring cell and 17 mixed melanomas (Figs. 1, 2 and 3). Melanocytomas belonged to the composite epithelioid (*n* = 3) or mixed (*n* = 4) subtypes. Twenty-one tumours (42%) were completely amelanotic; all but one of them (95%) were positive to S100, whereas 10 cases (48%) expressed Melan A, including the S100-negative tumour. The remaining tumours, including all the melanocytomas, had either a degree of pigmentation below (*n* = 18; 36%) or above (*n* = 11; 22%) 50%. Seventy-six percent of the mucosal tumours were achromic versus 24% of the cutaneous tumours (*P* < 0.001; Fisher’s exact test). Eighty-seven percent of spindle cell melanomas were amelanotic versus 25% of epithelioid or mixed types (*P* = 0.002; Fisher’s exact test).

Junctional activity, lymphocytic infiltrate and necrosis were observed in 34%, 38% and 16% of cases, respectively. Vascular invasion was identified in 7 tumours (14%) with epithelioid or balloon cell differentiation (Table 1).

**Table 1:** Relationship between proliferation indices (mitotic count and Ki-67 index) and clinicopathologic variables in 50 cases of feline non-ocular melanocytic tumors

Variables	Number of cases	Median mitotic count (range)	*P*	Median Ki-67 index (range)	*P*
Tumor location			0.396		0.984
skin	33	15 (0–153)		29% (1–78%)	
mucosae	17	15 (2–48)		22% (10–68%)	
Largest diameter^a^			0.052		0.014*
≤ 1.3 cm	28	10 (0–40)		18% (1–78%)	
> 1.3 cm	22	17 (3–153)		31% (14–77%)	
Morphological diagnosis			< 0.001*		< 0.001*
melanocytoma	7	2 (0–4)		6% (1–18%)	
melanoma	43	17 (1–153)		30% (6–78%)	
Prevalent histotype			0.002*		0.005*
spindle cell	8	28 (16–153)		38% (20–54%)	
others	42	12 (0–40)		25% (1–78%)	
Junctional activity			0.005*		0.026*
present	17	11 (0–27)		22% (1–61%)	
absent	33	21 (0–153)		32% (2–78%)	
Pigmentation			< 0.001*		< 0.001*
≥ 50%	11	3 (0–27)		8% (1–29%)	
< 50% or absent	39	17 (1–153)		31% (9–78%)	
Vascular invasion			0.353		0.565
present	7	19 (4–36)		32% (10–47%)	
absent	43	13 (0–153)		27% (1–78%)	
Lymphocytic infiltrate			0.299		0.14
present	19	17 (2–40)		31% (11–78%)	
absent	31	12 (0–153)		25% (1–68%)	
Necrosis			0.166		0.29
present	8	22 (4–153)		33% (10–71%)	
absent	42	13 (0–48)		27% (1–78%)	

*Significant

^a^Median value as cut-off

Median MC and Ki-67 index were 15 (range, 0–153) and 28% (range, 1–78%), respectively. These parameters were correlated (*P* < 0.001; *R* = 0.67; Spearman’s rank correlation). MC was significantly higher in tumours diagnosed as malignant, in spindle cell tumours, in those lacking junctional activity and in those with a percentage of pigmented cells below 50%. Ki-67 index was significantly higher in all of the above and in tumours larger than 1.3 cm (Table 1).

### Clinical course

Follow-up information was available for 33 cats (66%; with 21 cutaneous and 12 oral tumours). Eighteen (54%) underwent surgery; one received radiation therapy and 2 cats underwent a multimodal approach consisting of radiotherapy plus dose-intense chemotherapy (carboplatin, doxorubicin) and surgery plus metronomic chemotherapy (cyclophosphamide, thalidomide). In the cats receiving surgery, margins were histologically clean in 14 cases (74%) and infiltrated in 5 (26%). The remaining 12 cats (36%) only received palliative care.

At the end of the study, 10 cats (30%) were alive, after a median follow-up time of 140 days (95% CI, 64–401). Four cats (12%) had died for tumour-unrelated causes (chronic renal failure, *n* = 2; diabetes, *n* = 1; intestinal mast cell tumour, *n* = 1) after a median of 656 days (95% CI, 446–1175) and 19 cats (58%) had died of melanoma, with a median OS of 150 days (95% CI, 94–206).

For both MC and Ki-67 index, it was not possible to identify cut-off values to satisfactorily separate tumours with benign and aggressive biologic behaviour. When applying threshold values similar to those reported for canine melanoma (5 for MC and 20% for Ki-67 index), both were significantly associated with survival (Table 2).

**Table 2:** Relationship between overall survival (OS) and clinicopathological variables in 33 cases of feline non-ocular melanocytic tumors with available follow-up information

Variables	Number of cases	Median OS (95% CI)^c^	*P*
Tumor location			0.036*
Skin	21	197 (133–261)	
Mucosae	12	72 (48–96)	
Largest diameter^a^			0.006*
≤ 1.3 cm	14	689 (79–1299)	
> 1.3 cm	19	75 (11–139)	
Morphological diagnosis			0.109
Melanocytoma	3	Not reached	
Melanoma	30	156 (91–221)	
Prevalent histotype			0.014*
Spindle, balloon and signet ring cell	10	48 (0–115)	
Epithelioid	9	689 (0–1444)	
Mixed	14	156 (125–187)	
Junctional activity			0.412
Present	10	136 (101–171)	
Absent	23	197 (120–273)	
Pigmentation			0.015*
≥ 50%	8	Not reached	
< 50% or absent	25	119 (29–208)	
Vascular invasion			0.809
Present	6	176 (0–405)	
Absent	27	156 (77–235)	
Lymphocytic infiltrate			0.838
present	12	176 (39–313)	
absent	21	156 (92–219)	
Necrosis			0.681
present	7	197 (48–346)	
absent	26	156 (116–196)	
Mitotic count^b^			0.013*
≤ 5	9	689 (0–1451)	
> 5	24	119 (32–206)	
Ki-67 index^b^			0.036*
≤ 20%	10	689 (0–1460)	
> 20%	23	119 (34–204)	
Treatment			< 0.001*
Yes	21	689 (12–1366)	
No	12	59 (20–98)	

*CI* confidence interval

*Significant

^a^Median value

^b^Cutoff value based on data analysis and canine melanoma literature

^c^Days

Other variables significantly associated with shorter survival times included mucosal location, large tumour size, spindle, balloon or signet ring cell histotypes; less than 50% of pigmented neoplastic cells and lack of treatment (Table 2). Cats with clean surgical margin had a significant better outcome than cats with infiltrated margins (*P* < 0.001; log-rank test) On multivariable survival analysis, only tumour histotype and treatment administration retained prognostic significance (Table 3).

**Table 3:** Multivariable analysis of variables potentially related to overall survival (OS) in 33 cases of feline non-ocular melanocytic tumors with available follow-up information

Variables	Hazard ratio	95% CI	*P*
Mucosal location	2.26	0.71–7.19	0.165
Largest diameter > 1.3 cm^a^	3.46	0.7–17.06	0.128
Spindle-, balloon cell and signet ring histotypes	3.48	1.04–11.57	0.042*
Pigmentation < 50%	1.69	0.09–32.56	0.730
Mitotic count >5^b^	6.77	0.36–128.02	0.202
Ki-67 index > 20%^b^	0.14	0.01–2.05	0.152
Lack of treatment	4.22	1.18–15.05	0.027*

*CI* confidence interval

*Significant

^a^Median value

^b^cutoff value based on data analysis and canine melanoma literature

## Discussion

The majority of feline NOMs are reported to be malignant, but definitive information about clinical and histologic prognostic factors are lacking, mostly due to the low frequency of these neoplasms.

This is the second largest study on feline NOMs and the first investigating the prognostic relevance of Ki-67 in this species.

Although the observed age range was extremely wide, the majority of subjects were aged, confirming previous reports [1, 5, 10]. According to several authors, melanomas with primary auricular localization would affect younger subjects and be associated with a better outcome [1, 4–6]. This finding was not supported by our results, since only one of 7 cats with pinnal melanoma was under 10 years of age, and 3 cats out of 4 experienced rapid disease progression.

Apparently, a greater proportion of feline malignant melanomas arises in the skin, as compared with dogs, and the involved region has not a prominent role in the assessment of prognosis [1, 5]. Conversely, mucosal location seems to be associated with a worse prognosis, possibly due to a greater difficulty to obtain adequate local tumour control.

While being an acknowledged promoting factor for human and equine melanoma, ultraviolet (UV) light exposure was only hypothesized to play a role in feline cutaneous melanoma, mainly due to the common occurrence of these tumours on the head and ears [5, 6]. In the present study, the 50% of cats with cutaneous melanoma had no outdoor access, making the causative role of solar exposure less likely. Cats with orange, red, calico or silver coat are associated with a higher incidence of developing intraepidermal melanocytic hyperplasia (lentigo) on their lips, gums, eyelids and/or nose [11]. This is a benign condition that has not been reported to evolve to malignant melanoma, however, notably, 67% of the cats in this study were the above colours. Case-control studies could be helpful to clarify the role of UV radiation and hair color in feline NOMs.

The elective therapy for feline melanoma remains complete surgical excision. In the study by Chamel et al., (2016), cats undergoing surgery survived significantly longer than those receiving no treatment or medical treatment only. In the same study, complete surgical margins were not associated with a survival advantage [6]. In the present study, the median survival time of subjects receiving treatment was 3 times higher than those receiving a palliative treatment, but the presence of clean surgical margins was significantly correlated with a better prognosis.

Another previously reported negative prognostic factor which was confirmed by our results is the lack of melanin. The degree of pigmentation is a well acknowledged prognostic factor also in canine melanoma [9]. Indeed, the absence of melanin pigment could be associated with loss of differentiation and acquisition of a molecular phenotype with increased invasiveness and metastatic potential [2, 6].

Completely amelanotic melanomas are likely underdiagnosed in cats, due to difficulties in their identification. Depending on their morphological features, they can be misdiagnosed as undifferentiated carcinomas, sarcomas or round cell tumours (e.g. lymphoma, progressive histiocytosis, or atypical mast cell tumours). In this study, immunohistochemical positivity to at least one of Melan A and S100 was required for all amelanotic tumours, even in the presence of convincing morphological features. These are the only validated markers to confirm the melanocytic nature of a tumour in cats, however their limited diagnostic utility is acknowledged [6, 12]. As expected, less than 50% of amelanotic melanomas in this study were positive for Melan A, confirming the poor sensitivity of this marker. In contrast, almost all the amelanotic melanomas stained positively for S100, a highly sensitive but poorly specific marker.

In the literature, epithelioid, spindle cell, mixed, signet ring and balloon cell type of melanomas have been described in cats [10]. The relevance of tumour histotype in predicting the clinical behaviour of feline NOMs has been disputed, with some authors reporting a worse prognosis associated with epithelioid melanomas [4]. In the present study, spindle, signet ring and balloon cell histotypes were significantly associated with a poorer prognosis in comparison with epithelioid and mixed melanomas. Moreover, epithelioid tumours were characterized by lower proliferative activity and a higher degree of pigmentation.

Although Ki-67 index has been previously evaluated in 4 cats with ocular and extra-ocular melanomas [13], the prognostic relevance of proliferative activity has never been reliably assessed in this species. In the present study, Ki-67 index was significantly correlated with other prognostic variables, including tumour size, spindle cell histotype, lack of pigmentation and MC, and values greater than 20% were ultimately correlated with a worse outcome. However, the same statistical correlations were also observed for MC, suggesting that the immunohistochemical assessment of Ki-67 index in feline NOMs may not add much more to the plain assessment of MC on HE-stained sections.

Nevertheless, with both markers, we failed to identify a threshold value to satisfactorily identify tumours with a worse clinical outcome and, after adjustment with other clinicopathological variables in a multivariable model, prognostic significance was not retained. It must be reminded that the retrospective setting of this study resulted in not standardized staging procedures, treatment plans and follow-up schedules, making comparisons difficult. Almost all cats were treated in first opinion practices by a wide range of practitioners with different levels of experience, reflecting the clinical management of the majority of cats with this disease. Prospective studies are encouraged to assess the prognostic utility of the Ki-67 index on more cases with complete clinical staging, receiving gold standard treatments and with long term follow-up information.

## Conclusions

This study confirms a poor prognosis of the majority feline NOMs, although a certain degree of variability can be observed, with a better outcome observed for small, pigmented skin tumours removed with complete surgical margins. Contrarily to previous studies, the epithelioid morphology appears to be associated with a less malignant biologic behaviour. MC and Ki-67 index may contribute, with the aforementioned variables, to the prognostic assessment of feline NOMs. Reliable threshold values for both markers need to be identified in prospective, standardized studies.

## Methods

### Inclusion criteria

Lesions histologically diagnosed as feline non-ocular melanoma/melanocytoma or with melanoma among differential diagnoses were retrospectively retrieved from the archives of the pathology service of the Departments of Veterinary Medical Sciences (University of Bologna, Italy) and of Veterinary Sciences (University of Pisa, Italy), and from a private veterinary diagnostic laboratory (La Vallonea, Rho, Italy). Only primary tumours were considered for inclusion: local recurrences and nodal/distant metastases were removed from the selection.

Histologic sections from each case were reviewed for diagnosis confirmation. Amelanotic tumours were included only upon immunohistochemical positivity to at least one melanocytic marker with validated diagnostic utility in cats, including melan-A (1:400; A103 clone, Dako, Glostrup, Denmark) and S100 (Catalogue number: Z0311;1:2400; rabbit polyclonal, Dako) [12]. The immunohistochemical analysis was performed as part of the study at the Department of Veterinary Medical Sciences, University of Bologna.

### Histology

All samples for histologic examination were fixed in 10% neutered-buffered formalin, processed by routine methods, embedded in paraffin wax, sectioned at 4 μm and stained with haematoxylin and eosin (HE). The evaluated histologic features included morphological diagnosis (melanocytoma or malignant melanoma, according to the WHO guidelines) [14], prevalent histotype (epithelioid, spindle cell, balloon cell, signet ring cell or mixed), junctional activity, degree of pigmentation (more than 50% of pigmented cells, less than 50% or absent), vascular invasion, lymphocytic infiltrate, necrosis and mitotic count (MC). MC was assessed as the number of mitotic figures in a 2.37 mm^2^ area (10 fields with a 40× objective and a 10× ocular with a field number of 22 mm), according to the standards proposed by Meuten et al., 2016 [15]. The count was performed in 10 consecutive non-overlapping high-power fields (HPFs), starting from an area of high mitotic activity. Fields with necrosis or inflammation were skipped. All histologic evaluations were performed by consensus by two of the authors (SS and AR).

### Bleaching of melanin

The bleaching of melanin was performed on all the tumours with approximately more than 25% of pigmented cells before assessing MC and performing Ki-67 immunohistochemistry (IHC).

Briefly, 4 μm tissue sections were exposed to treatment with 2.5 g/L potassium permanganate (Merck, Darmstadt, Germany) for 5 min, followed by 50 g/L oxalic acid (Merck) for 5 min at room temperature.

To evaluate the effects of bleaching on tissue immunoreactivity, Ki-67 labelling was carried out on serial sections of an amelanotic melanoma, with and without bleaching. No differences in labelling intensity or distribution were observed, and it was therefore concluded that the bleaching method did not interfere with the assessment of tumour growth fraction.

### Ki-67 immunohistochemistry

Tumour sections were immunolabelled for Ki-67 by using a commercial anti-human primary antibody (MIB-1 clone, Dako) with validated reactivity in feline tissues [16].

Endogenous peroxidase activity was blocked by incubation for 30 mins with 0.3% hydrogen peroxide in methanol. For antigen retrieval, slides were microwaved in citrate buffer (pH 6.0) for 4 cycles of 5 mins, at 750 W. Sections were incubated overnight at 4 °C in a humid chamber with the primary antibody diluted 1:600 in a blocking solution (10% goat serum in phosphate-buffered saline). Binding sites of primary antibody were identified using a biotinylated goat anti-mouse secondary antibody (1:200 in blocking solution, Dako) with an incubation step of 30 min at room temperature. Sections were then incubated with a commercial streptavidin-biotin-peroxidase kit (Vectastain Elite ABC Kit, Vector Laboratories, Burlingame, CA, USA) and 3,3′-diaminobenzidine (DAB tablets, Diagnostic BioSystems, Pleasanton, CA, USA) was used as chromogen. Counterstain was performed with Papanicolaou’s haematoxylin.

Feline intestinal mucosa was used as positive control for MIB-1. Negative controls were obtained by omitting the primary antibody.

The evaluation of Ki-67 immunolabelling was performed by two authors (SS, MF), without knowledge of the case outcome. Five high-power (400×) fields selected within the areas of highest Ki-67 positivity were photographed. Areas with severe inflammation or necrosis were avoided. In every image, the number of neoplastic cell nuclei with positive labelling and the total number of neoplastic cells were assessed manually with a digital cell counter (ImageJ, National Institutes of Health, Bethesda, MD, USA). Ki-67 index was calculated as the mean percentage of labelled neoplastic cells in the 5 photographed fields.

### Clinical information

Patient records were reviewed to collect signalment, tumour location and tumour size (largest diameter).

Referring veterinarians and/or owners were contacted for additional information, including hair colour, living environment (indoor/outdoor), clinical presentation, presence of metastases, treatment, survival and patient status.

The availability of these data was not among inclusion criteria.

### Statistical analysis

Data were analysed by use of a commercial software program (SPSS Statistics v19, IBM, Armonk, NY, USA); *P* values ≤0.05 were considered significant.

When appropriate, data sets were tested for normality by use of the D’Agostino and Pearson omnibus normality test. Values were expressed as mean ± standard deviation in case of normal distribution, or as median with a range in case of non-normal distribution.

The relationships between the following variables were investigated: tumour location, tumour size, morphological diagnosis, prevalent histotype, junctional activity, degree of pigmentation, vascular invasion, lymphocytic infiltrate, necrosis, MC and Ki-67 index. The distributions of qualitative and quantitative variables were assessed by Fisher’s exact test and Student’s T/Mann-Whitney U test, respectively. The correlation between MC and Ki-67 index was evaluated by means of the Spearman’s rank correlation coefficient.

Overall survival (OS) was defined as the time (days) from the date of diagnosis to the last reported date on which the patient was seen alive. The patient status was recorded as alive, dead because of melanoma-unrelated causes or dead because of melanoma-related causes. Survival estimates are presented as medians with the corresponding 95% confidence intervals (95% CI).

The log-rank test was applied to compare survival distributions. Single variables analysed for prognostic relevance included tumour location, tumour size, morphological diagnosis, prevalent histotype, junctional activity, degree of pigmentation, vascular invasion, lymphocytic infiltrate, necrosis, MC, Ki-67 index and received treatment. Significant variables were further tested in a multivariable Cox proportional hazard model.

## Abbreviations

CI: Confidence interval
HE: Haematoxylin and eosin
HPF: High-power field
IHC: Immunohistochemistry
MC: Mitotic count
NOM: Non-ocular melanomas
OS: Overall survival
UV: Ultraviolet
